# Therapeutic and nutraceutical potentials of a brown seaweed *Sargassum fusiforme*

**DOI:** 10.1002/fsn3.1835

**Published:** 2020-08-20

**Authors:** Jian Liu, Sibusiso Luthuli, Yue Yang, Yang Cheng, Ya Zhang, Mingjiang Wu, Jong‐il Choi, Haibin Tong

**Affiliations:** ^1^ College of Life and Environmental Science Wenzhou University Wenzhou China; ^2^ Department of Biotechnology and Bioengineering Chonnam National University Gwangju Korea

**Keywords:** antiangiogenic, antitumor, immunomodulatory, neurodegenerative, *Sargassum fusiforme*

## Abstract

*Sargassum fusiforme*, also known as Yangqicai (羊栖菜) in Chinese and *Hijiki* in Japanese, is a brown seaweed that grows abundantly along the rocky coastlines of Asian countries such as Japan, Korea, and China. The first use of *S. fusiforme* as a traditional Chinese medicinal plant was recorded in the Shennong Bencao Jing, dated 200 AD. It was referred to as Haizao (seaweed), renowned for treating Yinglu (tumor‐like induration), dysuria, and edema. Currently, it is commonly used in traditional cuisine as it is rich in dietary fiber and minerals such as calcium, iron, and magnesium. Owing to its health benefits, *S. fusiforme* remains popular in China, Korea, and Japan, as well as in the UK and in North America. Currently, there is a lack of research on *S. fusiforme*; thus, we review the therapeutic effects of *S. fusiforme*, such as anticancer, antiangiogenic, and antiviral effects, in vitro and in vivo as reported during the past two decades. This review may promote further research on the therapeutic uses of *S. fusiforme*. Furthermore, we discuss the processes and considerations involved in using drugs produced from marine sources.

## Graphical abstract

We review the therapeutic effects (anticancer, angiogenesis, and antiviral) in vitro and in vivo reported during the past two decades. This review would promote research on the therapeutic development of *Sargassum fusiforme*. Processes and considerations involved in using such drugs from marine sources are also presented.

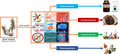

## 1 INTRODUCTION

Marine macroalgae (also referred to as seaweeds) belong to three major classes or phyla: Chlorophyceae (green algae), Rhodophyceae (red algae), and Phaeophyceae (brown algae). Each phylum comprises thousands of species, and vast chemodiversity has been described within and between these major algal groups (Baurain et al., 2010; Shannon & Abu, 2019). According to the Food and Agriculture Organization (FAO) database (2020), global aquaculture production of marine algae has more than tripled, from 10.6 million tons in 2000 to 32.4 million tons in 2018 (FAO, 2020). Since ancient times, seaweeds have been used as food products and as traditional medical agents, especially in Asian countries. Currently, there is interest in expanding the use of seaweeds and particularly in adding value to extracted components for a wide range of uses. Apart from its usage as a food source, seaweeds are also an excellent source of structurally diverse bioactive compounds with considerable pharmaceutical and biomedical potential for developing functional materials such as nutraceuticals and cosmeceuticals (Gupta & Abu, 2011). Over the past few years, seaweeds have been gaining considerable interest as sources of valuable functional metabolites. Every year, a substantial number of novel bioactive secondary metabolites are isolated from seaweeds, and these compounds are typically tested using various biological assays to assess their potential as anticancer, anti‐inflammatory, antiviral, antihypertensive, antibacterial, and antidiabetic agents (Fernando, Sanjeewa, Kim, Lee, & Jeon, 2018; Wijesekara, Pangestuti, & Kim, 2011; Wijesinghe & Jeon, 2012). Future efforts in the seaweed industry will help develop high‐value markets for functional foods, cosmeceuticals, nutraceuticals, and pharmaceuticals (Hafting et al., 2015).

Various natural products can be obtained from plants, animals, and microorganisms. Owing to the diversity of these products and lack of effective natural compounds from terrestrial sources, scientists began focusing their attention on developing drugs from marine sources (Senthilkumar, Manivasagan, Venkatesan, and Kim (2013)). *Sargassum fusiforme* (also referred to as *Hizikia* or *Hijiki*) is a macroalgal seaweed belonging to the class Phaeophyceae of the order Fucales (Cleveland, 2008). This species is typically distributed along the coastal areas of China, Korea, and Japan (Cong, Xiao, Liao, Dong, & Ding, 2014; Li, Fu, Duan, Xu, & Gao, 2018a). The market demand for *S. fusiforme* remains high because of its nutritional potential and its economic value in the pharmaceutical and manufacturing fields (Liu et al., 2018). Metabolites of *S. fusiforme* have been shown to possess antitumor (Chen, Nie, Yu, et al., 2012), antiviral (Sun et al., 2019), antiaging (Chen, He, et al., 2016), and anticoagulant properties (Sun et al., 2018). The unique characteristics of polysaccharides and bioactive low‐molecule compounds in this seaweed species are of particular interest, thus emphasizing the importance of further research in this area. Therefore, exploring the therapeutic and nutraceutic potential of this seaweed is required to increase public awareness and to efficiently utilize this sustainable natural resource. Usage of the bioactive metabolites of *S. fusiforme* in various applications as a source of functional ingredients is worthy of consideration. Hence, the objective of this review was to present current literature on *S. fusiforme*, highlighting its functional properties that may be important for promoting further use of this seaweed.

## 2 CHARACTERIZATION OF *S. FUSIFORME* POLYSACCHARIDES

Most studies on *S. fusiforme* have focused on polysaccharides, especially fucoidans; thus, we present data on sources, extraction methods, and analytical methods of fucoidans before describing their usage in in vitro and in vivo studies (Table 1). Fucoidans are associated with several bioactivities of *S. fusiforme*. Different methods of extraction and analysis of fucoidans have been reported. *Sargassum fusiforme* polysaccharides (SFPSs) mainly consist of a small portion of laminarin, alginic acid, fucoidan, and dietary fiber. Wang et al. (2009) reported that wild *S. fusiforme* contained 32.18% alginic acid (calculated as glucuronic acid), 2.40% fucoidan (calculated as fucose), and 0.54% laminarin (calculated as glucose). These three compounds occur at higher concentrations in wild *S. fusiforme* than in cultivated specimens (Fitton, Stringer, & Karpiniec, 2015; Wang et al., 2009).

Fucoidan is one of the most pharmacologically significant active compounds produced by *S. fusiforme*. The considerable heterogeneity of fucoidans entails that their molecular weight cannot be discretely determined; instead, they are characterized by a range of molecular weights, typically referred to as a scope, or by the “numerical average” or median peak weight (Fitton et al., 2015). Monosaccharide composition analysis of polysaccharides is crucial for research on polysaccharide structures and characteristics. SFPSs have been shown to predominantly consist of fucose, mannuronic acid, galactose, glucosamine, mannose, glucose, glucuronic acid, galacturonic acid, guluronic acid, rhamnose, and xylose (Wu, Jiang, Lu, Yu, & Wu, 2014). A structural assessment of fucoidans is typically conducted through partial degradation of methylations, gas chromatographical, mass spectrometry, and nuclear magnetic resonance spectroscopy (Cong et al., 2016; Hu et al., 2016). Fucose typically consists of α‐L‐fucose units connected by (1 → 4)‐ and (1 → 3)‐glucosidic bonds and is sulfated at the C‐2 and/or C‐3 and/or C‐4 positions. Depending on the species, its structure may comprise a linear backbone of α‐L‐fucopyranose residues with (1 → 3)‐ or alternating (1 → 3)‐/(1 → 4)‐glucosidic bond linkages (Li, Lu, Wei, & Zhao, 2008).

Bioactivity of polysaccharides depends on their chemical structure and molecular conformation. Polysaccharides have various bioactivities in solutions because they occur in diverse conformations including single, double, and triple helices (Anderson, Campbell, Harding, Rees, & Samuel, 1969; Norisuye, Yanaki, & Fujita, 1980; Saitô, Ohki, & Sasaki, 1977), polymers (Schlecht Pietsch, Wagner, & Anderson, 1994), disordered curls (Morris, Cutler, Ross Murphy, Rees, & Price, 1981), rod‐like structures (Jana, Gearheart, & Murphy, 2001), and spherical structures (Gu & Catchmark, 2012). However, the solution behavior and molecular conformation of *S. fusiforme* polysaccharides remain unclear. Using scanning electron microscopy, a recent study demonstrated that fucoidan extracts of *S. fusiforme* exhibited an inhomogeneous texture with a flaky or villi‐ and powder‐like structure (Liu et al., 2020).

Taken together, examining the relationship between chemical structure, chain conformation, and biological activity of *S. fusiforme* polysaccharides is of great importance for drawing further conclusions. The general relationship between the bioactivity and structure of *S. fusiforme* polysaccharides remains unclear, even though the bioactivity of SFPS has been extensively explored in the past decade.

## 3 APOPTOSIS INDUCTION AND ANTICANCER ACTIVITY

Apoptosis‐mediated cell death has become an extensively researched topic. Understanding disease‐associated apoptosis is essential as it can provide fundamental information regarding the etiology of the disease and may aid in choosing the optimal treatment. For example, cancer involves an imbalance between cell division and cell death, where cells that are supposed to die fail to receive the corresponding signals. Such processes can arise at any stage of the apoptotic pathway. Downregulation of the tumor suppressor gene *p53* leads to reduced apoptosis and enhanced growth and development of tumor cells (Bauer & Helfand, 2006), and inactivation of *p53*, regardless of the mechanism, has been associated with a number of human cancers (Iacopetta, 2003; Morton et al., 2010). The ability of malignant cells to evade apoptosis is a hallmark of cancer (Hanahan & Weinberg, 2011). Cancer cells show multiple characteristics; for example, they can evade cell cycle checkpoints and withstand exposure to cytotoxic agents (Letai, 2008). Therefore, causing targeted apoptosis is therapeutically important for treating cancers (Plati, Bucur, & Khosravi, 2011).

A study on the antitumor effects of SFPSs (299 kDa) on liver cancer was conducted by Fan et al. (2017) who used SFPSs that were mainly composed of d‐fucose, l‐xylose, d‐mannose, and d‐galactose, with a relative molar ratio of 5.9:1.0:2.3:2.2. Additionally, SFPSs contained 10.74% ester sulfate and 6.48% uronic acid. In this study, different dosages of *S. fusiforme* were orally administered to mice inoculated with human hepatocellular carcinoma cells HepG2. Their results showed antitumor effects of *S. fusiforme* when administered at high concentrations. SFPSs produced stimulatory effects on apoptosis in HepG2 cells by upregulating the expression of *Bax* and downregulating the expression of *Bcl‐2*. In a different study by Ji, Ji, and Yue (2014). SFPS‐B2 induced apoptosis of human gastric cancer cell line SGC‐7901 within 24 hr of administration. SFPS‐B2 facilitated activation of mitochondrial permeability transition pore, a mitochondrial transmembrane protein, and decreased the levels of matrix metalloproteinases (MMPs). Furthermore, increased activity of caspase‐9 and caspase‐3 was observed with downregulation of *Bcl‐2* expression of and upregulation of *Bax* expression, thus inducing the release of cytochrome c. Chen, Zhang, et al. (2017)) examined the proficiency of SFPSs in inducing apoptosis and evaluated their antitumor effects in human lung adenocarcinoma SPC‐A‐1 cell and its xenograft model. Their results showed that SFPSs prevented SPC‐A‐1 proliferation in vitro and tumor growth in vivo. Additionally, immunohistochemistry showed a decrease in tumor microvessel density as well as CD31 and vascular endothelial growth factor (VEGF)‐A expression levels. The results of such pro‐apoptotic tests indeed encourage further research on *S. fusiforme*, which has considerable potential in cancer treatment. With continuous accumulation of data, *S. fusiforme* may prove to be an important part of cancer therapy.

## 4 ANTIANGIOGENIC ACTIVITY

Angiogenesis is typically defined as growth from pre‐existing vasculature. Several preclinical and related studies have shown that angiogenesis plays a vital role in oncogenesis and metastasis; consequently, suppression of angiogenesis in tumor tissue may hold considerable treatment potential (Gimbrone, Leapman, Cotran, & Folkman, 1972; Graham, Rivers, Kerbel, Stankiewicz, & White, 1994). Paracrine stimulation of tumor cells is considered the main promotor of angiogenesis. Endothelial cells, once activated by this stimulus, will proliferate and migrate to form new vessels and blood flow. This process involves a variety of biomolecules. VEGF is one of the most effective and prominent angiogenic factors in tumor‐induced angiogenesis (Claffey & Robinson, 1996), and it was initially recognized for its capacity to initiate vascular permeability and induce growth of endothelial cells.

VEGF has recently been identified as a critical factor of neoplasm growth (Ferrara & Alitalo, 1999). Numerous tumors produce VEGF; clinically, inhibiting VEGF‐induced angiogenesis can significantly suppress tumor growth in vivo, which emphasizes the critical role of VEGF in neoplasm growth (Millauer, Shawver, Plate , Risaui, & Ullrich, 1994; Saleh, Stacker, & Wilks, 1996). Chen, Cong, et al. (2016) demonstrated that angiogenesis associated with VEGF‐A and proliferation of pulmonary cancer in vitro and in vivo can be inhibited by FP08S2, a sulfated fucoidan fraction from *S. fusiforme* (4.75 kDa) with sugar proportions of 36.6% fucose, 18.3% xylose, 7.0% mannose, 19.1% galactose, and 19.1% glucuronic acid in a graft model of human lung carcinoma cells. SFPSs also inhibited the expression of VEGF‐A in cancer cells and of VEGFR2, its receptor, in human umbilical vein endothelial cells. In addition, Chen, Cong, et al. (2016) observed that signaling pathways such as EGFR2, Erk, and VEGF may be blocked by FP08S2 in human microvascular endothelial cells‐1, thereby preventing growth and microvascular generation in a xenograft model of A549 cancer cells in nude rats. FP08S2 is a sulfated fucoidan of *S. fusiforme*, and owing to its remarkable antiangiogenic activity, it may indeed be the preeminent compound that inhibits lung cancer cell growth (Cong et al., 2016). Future studies should focus on following up previous research in this field to determine whether therapeutic avenues employing such polysaccharides can help cure malignant tumors or related growths that might eventually become malignant.

## 5 UV PROTECTION

The skin is the largest and most visible organ and is heavily affected by environmental factors; the skin, therefore, is the obvious choice for studying long‐term effects of UV‐induced aging and damage and the efficacy of protective measures (Ranadive, Menon, Shirwadkar, & Persad, 1989). Human skin is continuously exposed to diverse environmental stimuli including air, environmental pollutants, and solar UV radiation. UV radiation can cause physical changes, immunological changes such as inflammation and impaired wound healing, and DNA damage, thereby promoting cellular senescence and carcinogenesis (Dunaway et al., 2018).

*Sargassum fusiforme* reduce water loss through the skin and protect skin structure as well as immune organs, as observed in an in vivo mouse study by Ye et al. (2018), which demonstrated structural properties and protective effects of SFPSs against UV‐B radiation in hairless Kunming mice. The polysaccharide was composed of 58.10% ± 2.12% carbohydrate components, 1.01% ± 0.15% protein components, 9.85% ± 0.96% sulfate groups, and 17.66% ± 0.54% glucuronic acid, with an average molecular weight of 224 kDa. The monosaccharide composition of the SFPS was 43.2% l‐fucose, 3.5% rhamnose, 18.4% galactose, 1.5% glucose, 18.5% fructose, 5.9% xylose, and 9.0% mannose. This SFPS also alleviated UV‐B‐induced oxidative stress by increasing the activity of catalase and superoxide dismutase and reducing the levels of malondialdehyde, an end product of lipid peroxidation. Additionally, the levels of MMP‐1 and MMP‐9 were also suppressed as a consequence of the SFPS treatment. These results suggest that *S. fusiforme* can be used as a functional food supplement to achieve skin protection. Current climatic changes such as global warming may affect human skin health, thereby aggravating pre‐existing skin conditions. The above‐mentioned study provides an innovative perspective on skin protection and may contribute to the prevention of skin cancer. Another interesting finding of this study is that degradation of skin collagen and wrinkle formation were suppressed by the SFPS in the model group; this finding might be of significance to the cosmetics and beauty therapy industries.

## 6 INTESTINAL HOMEOSTASIS

The gut immune system and intestinal microbiota interact with each other, and this plays an essential role in maintaining homeostasis in relation to the intestinal system. However, these systems sometimes undergo modifications, especially in the elderly population, leading to the development of low‐grade inflammation, which may advance to more severe pathological conditions such as inflammatory bowel syndrome and colorectal cancer (Magrone & Jirillo, 2013). Chen, Yang, et al. (2017)) investigated whether *S. fusiforme* would improve microbiota in the small intestine by altering physiology and gut microbiota composition in mice. Administration of SFPSs alleviated reduced cytoprotective capacity in the small intestine by upregulating the Nrf2/ARE pathway. Furthermore, SFPSs to some degree remodeled the complete microbiota community structure of the small intestine.

Recently, Cheng et al. (2019) examined the composition of the intestinal microbiota and used *S. fusiforme* fucoidan (SFF), which facilitated modification of the gut microbiota while alleviating streptozotocin‐induced hyperglycemia in mice. A comparison of diabetic mice with mice that were gavaged with SFF for 6 weeks revealed that SFF attenuated and reversed pathological changes in cardiac and hepatic cells and reduced oxidative stress in diabetic mice caused by streptozotocin; these changes are linked to metabolic syndromes. Furthermore, SFF substantially altered intestinal microbiota in diabetic mice; it is plausible that SFF reduced the relative abundance of intestinal bacteria associated with diabetes, suggesting that SFF can mitigate the symptoms of diabetes (Cheng et al., 2019). *Sargassum fusiforme*, thus, appears to promote gut health and may help alleviate illnesses associated with metabolic syndromes such as insulin resistance, hypertension, hyperlipemia, type 2 diabetes mellitus, and cardiovascular diseases. The above‐mentioned studies spark interest as the study of gut microbiota and the effects of SFPSs on gut microbiota are comparatively new aspects. These studies explored a novel avenue of research which is of clinicians, and even though this research field is still in its infancy, it may be a stepping stone into the future.

## 7 ANTIOXIDANT BIOACTIVITY

Antioxidants are molecules that are sufficiently stable for donating electrons to aggressive free radicals, thereby neutralizing them and reducing potential damage. Antioxidants can delay and ameliorate cell damage, mainly by means of their free radical scavenging properties (Lü, Lin, Yao, & Chen, 2010). Such low‐molecular weight antioxidants can safely interact with free radicals and consequently terminate oxidative chain reactions before damage to essential structures occurs. Examples of antioxidants include glutathione, ubiquinol, and uric acid, which are produced during normal metabolism (Shi, Noguchi, & Niki, 1999). Other kinds of antioxidants are absorbed from the diet. A wide variety of enzymes in the body are responsible for scavenging free radicals; however, the standard micronutrient (vitamin) antioxidants—vitamin E (α‐tocopherol), vitamin C (ascorbic acid), and β‐carotene—cannot be synthesized in the human body and must therefore be supplied through the diet (Levine, Rumsey, Daruwala, Park, & Wang, 1999).

An in vitro antioxidant evaluation on carboxymethylated and hydroxamated degraded SFPSs demonstrated significant radical scavenging abilities and high total antioxidant activity of these polysaccharides (Li, Chen, et al., 2018). Chen, He, et al. (2016)) observed that SFPSs exert strong antioxidant activity in vivo, and SFPSs promoted Nrf2‐dependent cytoprotection by upregulating nuclear Nrf2 translocation mediated by the p21 and JNK‐dependent pathways. This suggests that SFPSs may decelerate aging by enhancing the Nrf2‐dependent antioxidant defense system.

Wang, Lee, et al. (2018)) examined antioxidant abilities of sulfated polysaccharides from Celluclast‐assisted extracts of *Hizikia fusiforme* (HFPS) or SFPSs, and observed strong antioxidant activity of HFPS, suggesting that HFPS exert photoprotective effects. The antioxidant effect was shown to occur through regulation of the NF‐κB, AP‐1, and MAPK signaling pathways in vitro, after a high fat diet. In a different study, HFPS showed considerable free radical clearance capacity, thereby offering protection against oxidative stress induced by H_2_O_2_ in Vero cells and zebrafish (Wang, Oh, et al., 2018). Liu et al. (2020) investigated effects of different extraction techniques on yield, molecular characteristics, chemical components, and antioxidant activity of *S. fusiforme* fucoidans, and the extraction technique indeed influenced conformational shifts and chain stiffness of fucoidan and affected solution conformation in different ways. Of note, *S. fusiforme* fucoidans consistently showed antioxidant activities, regardless of the method. Some studies indicated that certain marine‐sourced polysaccharides are less effective when extracted using other methods; however, these results on *S. fusiforme* are enticing and encouraging as they confirm that *S. fusiforme* can exert antioxidant bioactivities, irrespective of the processing method. The interplay of structural, chemical, and antioxidant characteristics remains to be elucidated, and exploring these properties of *S. fusiforme* would be beneficial for potential development of novel therapies in the future.

## 8 NEUROPROTECTIVE EFFECTS

Natural or “alternative” remedies for treating physical and psychiatric disorders have gained worldwide popularity. Further studies are required before these remedies can be accredited as safe and effective alternatives, particularly regarding neuropharmacology (Mischoulon, 2009). Zhen et al. (2015) examined anticonvulsant and antidepressant properties of fucosterol extracted from *S. fusiforme* in mice. Fucosterol significantly elevated the levels of dopamine, norepinephrine, and 5‐hydroxyindoleacetic acid metabolite in mouse brains and thus demonstrated that the effects of fucosterol may be attributed to the upregulation of neurotransmitters. The experiments were performed using maximal electroshock treatments, and fucosterol (20, 40, 100 mg/kg) was shown to possess anticonvulsant effects. At the specified dosages, fucosterol caused no neurotoxicity in mice. Fucosterol may be a therapeutic option for treating epilepsy‐related depression. In the fucosterol cohort (20 mg/kg), a significant increase was observed in the concentration of hippocampal brain‐derived neurotrophic factor, which plays a critical role in neuronal survival and growth and serves as a neurotransmitter modulator (Bathina & Das, 2015; Hu et al., 2016) used an *S. fusiforme* fucoidan termed SFPS65A, which comprises fucose, galactose, xylose, glucose, glucuronic acid, and mannose. SFPS65A improved cognitive dysfunction in mice treated with scopolamine, ethanol, and sodium nitrite to induce memory deficiency. Overall, the results indicate that *S. fusiforme* produces compounds which can be used for treating neurodegenerative disorders.

## 9 ANTI‐OSTEOARTHRITIC EFFECTS

Numerous elderly people have from osteoarthritis, which is a degenerative disease that has been associated with chronic health conditions and is one of the predominant causes of disability and pain in adults (Allen & Golightly, 2015). Osteoarthritis, a type of inflammation that affects the synovium and cartilage and leads to subchondral bone tissue degradation, is the most common form of arthritis (Cross et al., 2014), resulting in pain, stiffness, and complete failure of affected joints (Barve et al., 2007; Henrotin & Kurz, 2007; Krasnokutsky, Attur, Palmer, Samuels, & Abramson, 2008). Numerous studies indicated that joint degeneration due to osteoarthritis results from a combination of biochemical factors and mechanical stresses (Henrotin & Kurz, 2007; Krasnokutsky et al., 2008; Lee et al., 2015). Therefore, expression of tumor necrosis factor (TNF)‐α and inflammatory factors such as interleukin (IL)‐1β by chondrocytes as well as synovial cells occurs at advanced stages of osteoarthritis, and consequently, the levels of MMPs and some pro‐inflammatory factors such as prostaglandin E2 (PGE2), IL‐8, IL‐6, and nitric oxide increase (Krasnokutsky et al., 2008).

*Sargassum fusiforme* extracts have shown inhibitory effects on degenerative diseases such as osteoarthritis in vitro and in vivo. Primary cultures of rat chondrocytes and a rat model of osteoarthritis induced by monosodium iodoacetate were used by Lee et al. (2015) who showed that *S. fusiforme* extract (20% EtOH) increased cell survival. Additionally, anabolic factors were increased (genetic expression of collagen type I, II, and aggrecan), and catabolic factors were significantly inhibited. In in vivo studies where *S. fusiforme* extract was orally administered, anabolic factors were increased, and expression of the catabolic factors MMP‐3 and MMP‐7 and production of NO and PGE2 were substantially inhibited. These findings indicate that *S. fusiforme* ethanol extracts may be therapeutic agents of interest for treating degenerative osteoarthritis.

## 10 ANTIVIRAL ACTIVITY

The treatment of human immunodeficiency virus (HIV) type 1 presents major challenges for antiviral therapies, including treatment complications, drug toxicity and resistance, and high costs. Hence, novel compounds which may help overcome such limitations are urgently needed (Wu, Attele, Zhang, & Yuan, 2001). Paskaleva et al. (2006) found that an *S. fusiforme* extract prevented HIV replication during a postentry event in the virus' life cycle, that is, when infected macrophages serve as a bridge between the central and the peripheral nervous system through spreading HIV‐1 infection to microglia and astrocytes in the central nervous system (Imam, 2005). Treatment with 1 mg/ml *S. fusiforme* extract inhibited active replication of the microglial cell cultures and R5‐tropic viruses by 90% in primary human macrophages. In primary human astrocytes, the *S. fusiforme* extract significantly inhibited independent entry of infection in VSV/NL4‐3 cells (Paskaleva et al., 2014). In a different study, palmitic acid was isolated from *S. fusiforme* and was found to be a bioactive compound that can inhibit both X4‐ and R5‐tropic HIV entry into CD4 T cells (Paskaleva et al., 2008). Furthermore, bioactivity‐guided fractionation of an *S. fusiforme* mixture yielded the bioactive compound SP4‐2, which at 8 μg/ml was found to inhibit HIV‐1 infection by 86.9%, with an IC_50_ value of 3.7 μg/ml. This represents a 230‐fold enhancement of antiretroviral potency, compared to the whole extract (Paskaleva et al., 2008). Western medicine is predominant in the search for drugs to treat HIV infection; however, traditional Chinese medicine may also help identify candidate drugs for HIV treatment, including *S. fusiforme* compounds, which broadens the perspective on potential future cures (Guo et al., 2014).

## 11 ANTIBACTERIAL ABILITY

Seaweed has been shown to produce bioactive components which can inhibit growth of a variety of Gram‐positive and Gram‐negative bacteria, including pathogens (Liu, Heinrich, Myers, & Dworjanyn, 2012). Seaweed extracts have been employed as antibiotics, anthelmintics, remedies for cough, hypertension, and diarrhea, for treating cancer, and for prophylaxis (Siddhanata, Ramavat, & Chauhan, 1991). Thus, the bioactive properties of seaweed have attracted research interest. For example, El, Ali, and El (2016). compared inhibitory effects of compounds methanol, diethyl ether, chloroform extracts, and ethanol with those of extracts from *Sargassum vulgare,* Rhodophyta (*Ceramium rubrum*), Phaeophyta (*Padina pavonica*), and *S. fusiforme* collected from the Red Sea, Egypt, and suggested that *S. fusiforme* extract produced the strongest inhibitory effects. These extracts were evaluated for their antibacterial capacity against 10 multidrug‐resistant clinical isolates of Gram‐positive and Gram‐negative strains. Among the extracts, a 100‐μl diethyl ether extract from *S. fusiforme* showed the highest inhibitory capacity. These results demonstrate that *S. fusiforme* is a promising agent against multidrug‐resistant bacteria that represent a major challenge in clinical settings.

## 12 IMMUNOMODULATORY EFFECTS

Clinical medicine focuses on treating malignant diseases by using chemotherapeutic drugs. However, such drugs are immunosuppressive, that is, the drugs weaken the patient's immune responses even though the intention is to improve the chance of survival (Corper & Rensch, 1921). Hence, there is a need for immunomodulators or other therapeutic alternatives to assist with ameliorating such adverse effects of chemotherapy (Harris, Sengar, Stewart, & Hyslop, 1976). These alternatives should also accelerate recovery in terms of boosting the immune system after chemotherapeutic treatment. In light of this, a group of scientists started to assess the efficacy of *S. fusiforme* as an immunomodulator (Hu et al., 2014).

Immunomodulators are intrinsic and extrinsic substances that regulate or modify the scope, type, duration, or capacity of the immune response (Rasmussen & Arvin, 1982). Diet may affect maintenance of immune homeostasis (Burns & Goodwin, 2004; Derhovanessian, Solana, Larbi, & Pawelec, 2008; Gardner & Murasko, 2002; Gorczynski & Terzioglu, 2008; Lebish & Moraski, 1987). Chen, Nie, Fan, et al. (2012)) reported that *S. fusiforme* can be an efficacious immune‐potentiating supplement and that it can be used as an alternative to reduce immunosuppression induced by chemotherapy; therefore, it may be used as an immunostimulant in food and pharmaceutical industries. This conclusion was supported by results indicating that SFPSs promoted proliferation of splenic lymphocytes and secretion of cell factors (IFN‐γ, IL‐2, and IL‐6) in immunocompromised mice treated with cyclophosphamide. SFPSs significantly increased the spleen index. In line with a previous study (Wang et al., 2013), administration of SFPSs to immunocompromised mice (subjected to 200 mg/kg cyclophosphamide) showed immunomodulatory effects. This was confirmed by increased spleen indices and significant improved numbers of jejunal intraepithelial lymphocytes (IELs) and goblet cells. Goblet cells play an important role in lubrication (secretion) and protection against pathogens that are in contact with the intestinal lumen; (Deplancke & Gaskins, 2001; Foong & Bornstein, 2009; Gwynne, Ellis, Sjövall, & Bornstein, 2009). IELs, which are in close contact with enterocytes, are therefore activated in response to a number of pathological conditions that may affect the intestines (Chott et al., 1997; Kapp, Kapp, McKenna, & Lake, 2004). Chen et al. (2018) purified and characterized a novel fraction of SFPS, SFP‐F2, and showed that it exerted immune‐enhancing effects by inducing the CD14/IKK/NF‐κB and P38/NF‐κB signaling pathways in mice. Moreover, SFP‐F2 increased the production of cytokines including TNF‐α, IL‐1β, and IL‐6 in RAW264.7 cells. These effects can play a vital role in fine‐tuning the responses of various components of the immune system. Hence, *S. fusiforme* contains promising immunotherapeutic agents.

## 13 CONCLUSIONS AND FUTURE PERSPECTIVES

Increasing scientific research on marine algae as potential drugs suggests that natural products from seaweeds are a precious resource of bioactive ingredient. *Sargassum fusiforme* shows various bioactivities including antitumor, immunomodulatory, antiviral, and antioxidant effects. Although extensive biological activities of its compounds have been demonstrated with promising preclinical results, applications in clinical practice remain limited. Important questions in this field are yet to be answered; thus, it is necessary to progress from correlation studies to causation studies and from animal to human studies. As revealed in many studies, *S. fusiforme* produces various remarkable active metabolites. Among the compounds isolated from *S. fusiforme*, polysaccharides have received considerable attention in the scientific community due to their potential health‐promoting effects. Nevertheless, the relationship between structural features, solution behavior, space conformation, and bioactivity is still unclear. In future studies, structure–function relationships of SFPSs need to be systematically examined in depth. Furthermore, relationships between biological activities of SFPSs and their effects on gut microbiota are of considerable interest.

In the present review, we focused on bioactive properties of polysaccharides isolated from *S. fusiforme* in relation to their health‐promoting effects and to update bioactive properties of bioactive low‐molecular weight compounds isolated from this seaweed. Therefore, our review may help increase awareness of bioactive properties of *S. fusiforme* to utilize it as a valuable source for therapeutic and nutraceutical applications.

**TABLE 1:** Polysaccharides isolated from *S. fusiforme*

Type of polysaccharides	Chemical composition				Monosaccharide composition (weight ratio)	Molecular weight (kDa)	Model	Result	Reference
	Total carbohydrate (%)	Uronic acid (%)	Sulfate (%)	Protein (%)					
Crude polysaccharides	ND	6.48	10.74	ND	Fuc:Xyl:Man:Gal = 5.9:1.0:2.3:2.2	299	Human hepatocellular carcinoma cells (HEPG2)	SFPS had remarkable cytotoxicity to HepG2 cells in vitro and showed significant inhibition of tumor growth in mice	Fan et al. (2017)
Crude polysaccharides	ND	ND	ND	ND	ND	ND	Human gastric cancer cell line SGC−7901	SFPS‐B2 can induce human gastric cancer cell line SGC−7901 apoptosis	Ji et al. (2014)
Crude polysaccharides	ND	ND	ND	ND	ND	ND	Human lung adenocarcinoma SPC‐A−1 cell and its xenograft model	SFPS inhibited the tube formation of HUVECs and the tumor angiogenesis	Chen, Zhang, et al. (2017)
Fucoidan	16.8	34.6	20.8	ND	Fuc:Xyl:Man:Gal:GlcA = 36.6:18.3:7.0:19.1:19.1	47.5	Human microvascular endothelial cells (HMEC−1) A549 cancer cell xenograft in nude mice	Fucoidan showed remarkable antiangiogenic activity via blocking VEGF signaling	Chen, Cong, et al. (2016)
Crude polysaccharides	58.10 ± 2.12	17.66 ± 0.54	9.85 ± 0.96	1.01 ± 0.15	Fuc:Xyl:Man:Gal:Rha:Glc:Fru = 28.8:3.9:6.0:12.3:2.3:1.0:12.3	224	Hairless Kunming mice	SFPS reduced skin water loss and protected immune organs and skin structure in mice.	Ye et al. (2018)
Crude polysaccharides	ND	ND	ND	ND	ND	ND	Male ICR mice	Dietary SFPS may promote health of the small intestine, consequently allowing for healthy aging	Chen, Yang, et al. (2017)
Fucoidan	68.33	ND	14.55	4.13	Fuc:Xyl:Man:Gal:Rha:Glc = 16.7:1.1:1.3:6.2:1.0:1.6	205.8	Streptozotocin‐induced diabetic mice	Fucoidan has great potential as an adjuvant therapy strategy for diabetes	Cheng et al. (2019)
Crude polysaccharides	97.9	51.1	9.2	ND	Fuc:Xyl:Man:Gal:Rha:GlcA:Glc:ManA:GulA = 28.9:5.2:9.1:5.3:3.8:1.0:8.8:38.9	75	Chronic aging mice middle aged male ICR mice	SFPS may decelerate the aging process by enhancing Nrf2‐dependent cytoprotection	Chen, He, et al. (2016)
Crude polysaccharides	55.05 ± 0.09	ND	7.78 ± 0.23	ND	Fuc:Xyl:Gal:Glc = 9.0:2.9:3.9:1.0	ND	Vero cells and zebrafish	SFPS exerts strong antioxidant activity and UV protective effects	Wang, Oh, et al. (2018)
Fucoidan	WSFF: 63.53 ASFF: 67.43 CSFF: 72.90	WSFF: 8.30 ASFF: 4.47 CSFF: 6.82	WSFF: 14.79 ASFF: 3.40 CSFF: 13.12	WSFF: 4.70 ASFF: 0.92 CSFF: 2.81	Fuc:Xyl:Man:Gal:Rha:Glc:GlcA:GalA = WSFF: 46.9:6.28:15.8:20.2:0.7:2.1:8.1:0; ASFF: 38.0:5.8:1.8:37.9:14.2:2.0:0:0.2; CSFF: 61.4:4.0:5.8:19.1:0.9:3.6:2.0:3.1	WSFF: 65.34 ASFF: 26.63 CSFF: 69.15	DPPH and hydroxyl radical scavenging assays	Fucoidans showed different antioxidant capacities due to their distinct physicochemical characteristics	Liu et al. (2020)
Crude polysaccharides	ND	ND	ND	ND	ND	ND	ICR mice	SFPS showed pronounced antioxidant activities	Wang et al. (2013)
Crude polysaccharides	62.9	14.7	27.7	0.4	Fuc:Xyl:Man:Gal:GalA = 80.6:3.0:2.4:13.3:0.7	24	Raw 264.7 cell	SFPS enhanced immune responses in RAW264.7 cells	Chen et al. (2018)
Fucoidan	65.03	61.31	2.3	5.76	Fuc:Man:Gal:Glc:GalA = 2.82:1.15:3.04:1:6.51	11	ICR mice	Fucoidan reversed immunosuppression in CTX‐treated mice	Hu et al. (2014)
Fucoidan	67.5	41.04	17.5	5.22	Fuc:Xyl:Man:Gal:Glc:GlcA = 19.23:6.64:2.57:9.58:1:6.52	90	Male ICR mice	Fucoidan improved cognitive dysfunction in mice	Hu et al. (2016)

Note

ND, not determined. SFPS symbolizes *Sargassum fusiforme* polysaccharides.

## CONFLICT OF INTEREST

The authors declare no conflict of interest.
